# Supplementary material for: The role of alien species on plant-floral visitor network structure in invaded communities
Source: PLoS One. 2019 Nov 8;14(11):e0218227. doi: 10.1371/journal.pone.0218227 (PMC6839871; doi:10.1371/journal.pone.0218227)
Supplement: S1 Table — List of recorded plant species in the study area. NE = Native Sppecies, AS = Alien Species. (DOCX) [file pone.0218227.s001.docx]

TS1 List of recorded plant species in the study area. NE= Native Sppecies, AS= Alien Sppecies.

| **Families** | **Species** | **Code** | **Status** | **Life form** |
| --- | --- | --- | --- | --- |
| Aizoaceae | *Sesuvium portulacastrum* (L.) L. | Sepo | NE | Herbaceous |
| Amaranthaceae | *Alternanthera microcephala* (Moq.) Schinz | Almi | AS | Herbaceous |
|  | *Amaranthus greggii* S. Watson.  (=*Amaranthus dubiuis*) | Amgr | AS | Herbaceous |
|  | Atriplex tampicensis Standl. | Atta | NE | Herbaceous |
|  | *Blutaparon vermiculare* (L.) Mears. | Blve | NE | Herbaceous |
|  | *Suaeda linearis* (Elliott) Moq. | Suli | NE | Herbaceous |
| Amaryllidaceae | *Hymenocallis littoralis* (Jacq.) Salisb. | Hyli | NE | Herbaceous |
| Asteraceae | *Bidens pilosa* L. | Bipi | AS | Herbaceous |
|  | *Flaveria linearis* Lag. | Flli | NE | Herbaceous |
|  | *Melanthera nivea* (L.) Small.  (=*Melanthera asppera*) | Meni | AS | Herbaceous |
| Brassicaceae | *Cakile edentula* (BigeloDW) Hook. | Caed | NE | Herbaceous |
| Combretaceae | *Conocarpus erectus* L. | Coer | NE | Tree |
| Commelinaceae | *Commelina elegans* Kunth | Coel | NE | Herbaceous |
| Convolvulaceae | *Ipomoea pes-caprae* (L.) R. Br. | Ippe | NE | Vine |
| Crassulaceae | *Maytenus phyllanthoides* Benth. | Maph | NE | Shrub |
| Euphorbiaceae | *Croton punctatus* Jacq. | Crpu | NE | Herbaceous |
|  | *Euphorbia cyathophora* Mur. | Eucy | AS | Herbaceous |
|  | *Euphorbia mesembryanthemifolia* Jacq. | Eume | NE | Herbaceous |
| Fabaceae | *Canavalia rosea* (SDW.) DC. | Caro | NE | Vine |
| Goodeniaceae | *Scaevola plumieri* (L.) Vahl | Scpl | NE | Shrub |
| Malvaceae | *Malvaviscus arboreus* Cav. | Maar | NE | Shrub |
|  | *Waltheria rotundifolia* Schrank. | Waro | NE | Herbaceous |
| Nyctaginaceae | *Commicarpus scandens* (L.) Standl. | Cosc | AS | Herbaceous |
|  | *Okenia hypogaea* Schltdl. & Cham. | Okhy | NE | Herbaceous |
| Passifloraceae | *Passiflora foetida* L. | Pafo | AS | Vine |
| Solanaceae | *Lycium carolinianum* Walter | Lyca | NE | Herbaceous |
| Surianaceae | *Suriana maritima* L. | Suma | NE | Shrub |
| Verbenaceae | *Lantana involucrata* L. | Lain | AS | Shrub |
|  | *Phyla nodiflora* (L.) Greene | Phno | AS | Herbaceous |
| Zygophyllaceae | *Tribulus cistoidesT* L. | Trci | AS | Herbaceous |
