# Supplementary material for: The role of alien species on plant-floral visitor network structure in invaded communities
Source: PLoS One. 2019 Nov 8;14(11):e0218227. doi: 10.1371/journal.pone.0218227 (PMC6839871; doi:10.1371/journal.pone.0218227)
Supplement: S2 Table — 2 List of insect flower visitors recorded in each site in the study area. The number of visits and the percentage per site is shown. (DOCX) [file pone.0218227.s002.docx]

TS 2 List of insect flower visitors recorded in each site in the study area. The number of visits and the percentage per site is shown.

| **Playa Maya** |  |  |  |  |  |
| --- | --- | --- | --- | --- | --- |
| **Order** | **Family** | **Species/morphospecies** | **Code** | **Visits** | **Percentage** |
| Hymenoptera | Apidae | *Apis mellifera* | Am | 621 | 33.7 |
| Lepidoptera | Hespperiidae | *Cymaenes trebius* | Lp | 180 | 9.8 |
| Diptera | Bombyliidae | *Crysanthrax editilius* | B3 | 145 | 7.9 |
| Diptera | Bombyliidae | *Crysntrax cypris* | B5 | 126 | 6.8 |
| Hymenoptera | Vespidae | Vesppide16 | Av15 | 116 | 6.3 |
| Diptera | Bombyliidae | *Crysntrax disppar* | B7 | 109 | 5.9 |
| Diptera | Bombyliidae | Bombilide6 | B6 | 80 | 4.3 |
| Lepidoptera | Nymphalidae | *Junonia scenia* | Js | 68 | 3.7 |
| Diptera | Syrphidae | Syrphidae1 | S1 | 68 | 3.7 |
| Diptera | Bombyliidae | *Paravilla* spp | B4 | 54 | 2.9 |
| Diptera | Bombyliidae | *Crysanthrax disppar* | B2 | 45 | 2.4 |
| Diptera | Bombyliidae | Bombilide10 | B10 | 33 | 1.8 |
| Diptera | Bombyliidae | *Geron* *spp* | D3 | 29 | 1.6 |
| Lepidoptera | Pieridae | *Ascia spp* | Aspp | 27 | 1.5 |
| Hymenoptera | Megachilidae | *Megachile zaptlana* | Mh | 25 | 1.4 |
| Diptera | Bombyliidae | Bombilide22 | D12 | 15 | 0.8 |
| Lepidoptera | Pieridae | *Ascia monuste* | Amo | 13 | 0.7 |
| Diptera | Muscidae | Muscidae | M | 13 | 0.7 |
| Hymenoptera | Halictidae | *Lassoiglosum spp* | La | 12 | 0.7 |
| Diptera | Bombyliidae | Bombilide9 | B9 | 11 | 0.6 |
| Diptera | Bombyliidae | Bombilide11 | B11 | 8 | 0.4 |
| Hymenoptera | Vespidae | *Polistes* spp1 | C1 | 8 | 0.4 |
| Lepidoptera | Pieridae | *Ascia josephina* | Aj | 7 | 0.4 |
| Diptera | Bombyliidae | *Exoprosa spp* | B1 | 6 | 0.3 |
| Diptera | Bombyliidae | Bombilide15 | D4 | 5 | 0.3 |
| Hymenoptera | Vespidae | *Micodynerus spp* | Av16 | 3 | 0.2 |
| Lepidoptera | Hespperiidae | Hespperidae | L5 | 3 | 0.2 |
| Hymenoptera | Vespidae | *Polistes nasidens* | Pn | 3 | 0.2 |
| Hymenoptera | Vespidae | Vesppide13 | Av12 | 2 | 0.1 |
| Diptera | Bombyliidae | Bombilide12 | D1 | 2 | 0.1 |
| Hymenoptera | Megachilidae | Megachilide1 | Mm | 2 | 0.1 |
| Hymenoptera | Scoliidae | Scoliidae1 | Av7 | 1 | 0.1 |
| Hymenoptera | Vespidae | Vesppide9 | Av8 | 1 | 0.1 |
| Lepidoptera | Nymphalidae | *Danaus erisimus* | Dae | 1 | 0.1 |
| Lepidoptera | Lycaenidae | Lycaenidae4 | L10 | 1 | 0.1 |
| Lepidoptera | Nymphalidae | Nymphalide1 | L2 | 1 | 0.1 |
| **Chapo 1** |  |  |  |  |  |
| Hymenoptera | Apidae | *Apis mellifera* | Am | 1841 | 68.9 |
| Lepidoptera | Hespperiidae | *Cymaenes trebius* | Lp | 178 | 6.7 |
| Diptera | Bombyliidae | *Crysanthrax editilius* | B3 | 154 | 5.8 |
| Hymenoptera | Vespidae | Polistes spp1 | C1 | 85 | 3.2 |
| Diptera | Bombyliidae | *Crysntrax cypris* | B5 | 76 | 2.8 |
| Diptera | Syrphidae | Syrphidae1 | S1 | 72 | 2.7 |
| Hymenoptera | Vespidae | *Polistes* spp2 | Pol | 48 | 1.8 |
| Hymenoptera | Halictidae | *Lassoiglosum spp* | La | 30 | 1.1 |
| Diptera | Bombyliidae | Bombilide6 | B6 | 23 | 0.9 |
| Hymenoptera | Vespidae | Vesppide16 | Av15 | 18 | 0.7 |
| Diptera | Bombyliidae | *Geron spp* | D3 | 17 | 0.6 |
| Hymenoptera | Vespidae | *Zethus spp* | Av9 | 16 | 0.6 |
| Hymenoptera | Megachilidae | *Megachile zaptlana* | Mh | 16 | 0.6 |
| Lepidoptera | Pieridae | *Ascia monuste* | Amo | 13 | 0.5 |
| Hymenoptera | Vespidae | *Micodynerus spp* | Av16 | 12 | 0.4 |
| Diptera | Bombyliidae | *Crysntrax disppar* | B7 | 12 | 0.4 |
| Lepidoptera | Nymphalidae | *Junonia scenia* | Js | 11 | 0.4 |
| Diptera | Bombyliidae | Bombilide11 | B11 | 10 | 0.4 |
| Diptera | Bombyliidae | Bombilide22 | D12 | 6 | 0.2 |
| Diptera | Bombyliidae | Bombilide18 | D7 | 6 | 0.2 |
| Diptera | Bombyliidae | *Paravilla spp* | B4 | 5 | 0.2 |
| Lepidoptera | Pieridae | *Ascia josephina* | Aj | 4 | 0.1 |
| Hymenoptera | Vespidae | Vesppide13 | Av12 | 4 | 0.1 |
| Hymenoptera | Vespidae | Vesppide3 | Av2 | 4 | 0.1 |
| Hymenoptera | Scoliidae | Scoliidae1 | Av7 | 3 | 0.1 |
| Hymenoptera | Vespidae | Vesppide4 | Av3 | 2 | 0.1 |
| Lepidoptera | Riodinidae | Rionidae3 | L12 | 2 | 0.1 |
| Lepidoptera | Pieridae | *Ascia spp* | Aspp | 1 | 0 |
| Hymenoptera | Vespidae | Vesppide11 | Av10 | 1 | 0 |
| Lepidoptera | Nymphalidae | *Agraullis vanillae* | Ava | 1 | 0 |
| Lepidoptera | Nymphalidae | *Danaus erisimus* | Dae | 1 | 0 |
| **Chapo 2** |  |  |  |  |  |
| Hymenoptera | Apidae | *Apis mellifera* | Am | 1845 | 69.9 |
| Hymenoptera | Vespidae | *Polistes* spp1 | C1 | 188 | 7.1 |
| Hymenoptera | Megachilidae | *Megachile zaptlana* | Mh | 85 | 3.2 |
| Lepidoptera | Hespperiidae | *Cymaenes trebius* | Lp | 79 | 3 |
| Hymenoptera | Vespidae | *Micodynerus spp* | Av16 | 67 | 2.5 |
| Lepidoptera | Pieridae | *Ascia monuste* | Amo | 59 | 2.2 |
| Diptera | Bombyliidae | Bombilide6 | B6 | 45 | 1.7 |
| Hymenoptera | Halictidae | *Lassoiglosum spp* | La | 34 | 1.3 |
| Lepidoptera | Nymphalidae | *Danaus erisimus* | Dae | 21 | 0.8 |
| Diptera | Bombyliidae | *Crysntrax disppar* | B7 | 20 | 0.8 |
| Diptera | Bombyliidae | *Geron spp* | D3 | 20 | 0.8 |
| Hymenoptera | Scoliidae | Scoliidae1 | Av7 | 17 | 0.6 |
| Diptera | Bombyliidae | *Crysanthrax editilius* | B3 | 17 | 0.6 |
| Lepidoptera | Pieridae | *Ascia spp* | Aspp | 16 | 0.6 |
| Lepidoptera | Pieridae | *Ascia josephina* | Aj | 14 | 0.5 |
| Hymenoptera | Vespidae | *Zethus spp* | Av9 | 14 | 0.5 |
| Hymenoptera | Vespidae | Vesppide3 | Av2 | 13 | 0.5 |
| Lepidoptera | Nymphalidae | *Agraullis vanillae* | Ava | 12 | 0.5 |
| Diptera | Bombyliidae | *Crysanthrax disppar* | B2 | 10 | 0.4 |
| Diptera | Bombyliidae | *Crysntrax cypris* | B5 | 10 | 0.4 |
| Lepidoptera | Pieridae | *Prysitas spp* | L1 | 9 | 0.3 |
| Hymenoptera | Vespidae | *Polistes nasidens* | Pn | 6 | 0.3 |
| Hymenoptera | Vespidae | Vesppide13 | Av12 | 5 | 0.2 |
| Hymenoptera | Vespidae | Vesppide6 | Av5 | 5 | 0.2 |
| Diptera | Bombyliidae | Bombilide22 | D12 | 5 | 0.2 |
| Lepidoptera | Nymphalidae | *Junonia scenia* | Js | 5 | 0.2 |
| Diptera | Bombyliidae | *Paravilla spp* | B4 | 4 | 0.2 |
| Diptera | Bombyliidae | Bombilide15 | D4 | 4 | 0.2 |
| Diptera | Syrphidae | Syrphidae1 | S1 | 4 | 0.2 |
| Hymenoptera | Vespidae | Vesppide16 | Av15 | 2 | 0.1 |
| Hymenoptera | Vespidae | *Polistes* spp2 | Pol | 2 | 0.1 |
| Diptera | Bombyliidae | Bombilide11 | B11 | 1 | 0.1 |
| Lepidoptera | Riodinidae | Rionidae3 | L12 | 1 | 0.1 |
| Diptera | Muscidae | Muscidae | M | 1 | 0.1 |
| Hymenoptera | Megachilidae | *Megachilide*1 | Mm | 1 | 0.1 |
| **Sisal** |  |  |  |  |  |
| Hymenoptera | Apidae | *Apis mellifera* | Am | 2911 | 87.3 |
| Lepidoptera | Pieridae | *Ascia josephina* | Aj | 170 | 5.1 |
| Lepidoptera | Pieridae | *Ascia monuste* | Amo | 30 | 0.9 |
| Lepidoptera | Pieridae | *Ascia spp* | Aspp | 23 | 0.7 |
| Hymenoptera | Vespidae | *Micodynerus spp* | Av16 | 22 | 0.7 |
| Hymenoptera | Vespidae | Vesppide3 | Av2 | 21 | 0.6 |
| Hymenoptera | Vespidae | Vesppide4 | Av3 | 17 | 0.5 |
| Hymenoptera | Vespidae | Vesppide5 | Av4 | 16 | 0.5 |
| Hymenoptera | Vespidae | Vesppide13 | Av12 | 14 | 0.4 |
| Hymenoptera | Vespidae | *Zethus spp* | Av9 | 14 | 0.4 |
| Hymenoptera | Vespidae | Vesppide12 | Av11 | 12 | 0.4 |
| Diptera | Bombyliidae | *Crysanthrax editilius* | B3 | 11 | 0.3 |
| Diptera | Bombyliidae | *Crysntrax cypris* | B5 | 10 | 0.3 |
| Diptera | Bombyliidae | Bombilide10 | B10 | 9 | 0.3 |
| Diptera | Bombyliidae | Bombilide11 | B11 | 7 | 0.2 |
| Hymenoptera | Vespidae | *Polistes* spp1 | C1 | 6 | 0.2 |
| Hymenoptera | Vespidae | *Polistes* spp 3 | C2 | 6 | 0.2 |
| Diptera | Bombyliidae | *Geron spp* | D3 | 6 | 0.2 |
| Diptera | Bombyliidae | Bombilide16 | D5 | 6 | 0.2 |
| Lepidoptera | Nymphalidae | *Junonia scenia* | Js | 5 | 0.1 |
| Lepidoptera | Riodinidae | Riodinide1 | L4 | 5 | 0.1 |
| Lepidoptera | Lycaenidae | Lycaenidae4 | L10 | 4 | 0.1 |
| Lepidoptera | Riodinidae | Rionidae3 | L12 | 3 | 0.1 |
| Hymenoptera | Halictidae | *Lassoiglosum spp* | La | 2 | 0.1 |
| Lepidoptera | Hespperiidae | *Cymaenes trebius* | Lp | 2 | 0.1 |
| Diptera | Syrphidae | Syrphidae1 | S1 | 2 | 0.1 |
| Diptera | Syrphidae | Syrphidae2 | S2 | 1 | 0.1 |
| **Telchac** |  |  |  |  |  |
| Hymenoptera | Apidae | *Apis mellifera* | Am | 479 | 44.3 |
| Lepidoptera | Pieridae | *Ascia monuste* | Amo | 106 | 9.8 |
| Hymenoptera | Megachilidae | *Megachile zaptlana* | Mh | 67 | 6.2 |
| Diptera | Bombyliidae | *Exoprosa spp* | B1 | 48 | 4.4 |
| Lepidoptera | Hespperiidae | *Cymaenes trebius* | Lp | 41 | 3.8 |
| Diptera | Bombyliidae | Bombilide10 | B10 | 39 | 3.6 |
| Diptera | Bombyliidae | *Geron spp* | D3 | 33 | 3 |
| Diptera | Bombyliidae | Bombilide15 | D4 | 30 | 2.8 |
| Hymenoptera | Halictidae | *Lassoiglosum spp* | La | 29 | 2.7 |
| Lepidoptera | Pieridae | *Ascia spp* | Aspp | 26 | 2.4 |
| Hymenoptera | Vespidae | *Polistes* spp1 | C1 | 24 | 2.2 |
| Diptera | Syrphidae | Syrphidae1 | S1 | 22 | 2 |
| Lepidoptera | Lycaenidae | Lycaenidae4 | L10 | 16 | 1.5 |
| Lepidoptera | Pieridae | *Ascia josephina* | Aj | 15 | 1.4 |
| Diptera | Bombyliidae | *Crysntrax disppar* | B7 | 12 | 1.1 |
| Lepidoptera | Hespperiidae | Hespperidae | L8 | 12 | 1.1 |
| Diptera | Bombyliidae | *Crysanthrax editilius* | B3 | 11 | 1 |
| Diptera | Bombyliidae | *Paravilla spp* | B4 | 11 | 1 |
| Lepidoptera | Nymphalidae | Nymphalide1 | L2 | 10 | 0.9 |
| Hymenoptera | Vespidae | Vesppide12 | Av11 | 9 | 0.8 |
| Hymenoptera | Vespidae | Vesppide13 | Av12 | 6 | 0.6 |
| Diptera | Bombyliidae | Bombilide11 | B11 | 6 | 0.6 |
| Lepidoptera | Nymphalidae | *Agraullis vanillae* | Ava | 4 | 0.4 |
| Hymenoptera | Vespidae | Vesppide11 | Av10 | 3 | 0.3 |
| Diptera | Bombyliidae | Bombilide8 | B8 | 3 | 0.3 |
| Diptera | Bombyliidae | Bombilide20 | D9 | 3 | 0.3 |
| Lepidoptera | Riodinidae | Riodinide2 | L13 | 3 | 0.3 |
| Hymenoptera | Vespidae | *Micodynerus spp* | Av16 | 2 | 0.2 |
| Diptera | Bombyliidae | Bombilide6 | B6 | 2 | 0.2 |
| Diptera | Bombyliidae | Bombilide9 | B9 | 2 | 0.2 |
| Lepidoptera | Pieridae | *Prysitas spp* | L1 | 2 | 0.2 |
| Lepidoptera | Riodinidae | Rionidae3 | L12 | 2 | 0.2 |
| Hymenoptera | Scoliidae | Scoliidae 2 | Av14 | 1 | 0.1 |
| Diptera | Bombyliidae | *Crysntrax cypris* | B5 | 1 | 0.1 |
| Diptera | Muscidae | Muscidae | M | 1 | 0.1 |
| Hymenoptera | Vespidae | *Polistes* spp2 | Pol | 1 | 0.1 |
| **Chabiahu** |  |  |  |  |  |
| Hymenoptera | Apidae | *Apis mellifera* | Am | 917 | 68.6 |
| Lepidoptera | Pieridae | *Ascia monuste* | Amo | 68 | 5.1 |
| Lepidoptera | Pieridae | *Ascia spp* | Aspp | 59 | 4.4 |
| Diptera | Bombyliidae | *Geron spp* | D3 | 42 | 3.1 |
| Hymenoptera | Halictidae | *Lassoiglosum spp* | La | 42 | 3.1 |
| Hymenoptera | Vespidae | *Micodynerus spp* | Av16 | 35 | 2.6 |
| Lepidoptera | Hespperiidae | *Cymaenes trebius* | Lp | 28 | 2.1 |
| Diptera | Bombyliidae | Bombilide17 | D6 | 19 | 1.4 |
| Hymenoptera | Vespidae | *Polistes* spp1 | C1 | 18 | 1.3 |
| Lepidoptera | Nymphalidae | *Junonia scenia* | Js | 15 | 1.1 |
| Lepidoptera | Nymphalidae | *Danaus erisimus* | Dae | 14 | 1 |
| Diptera | Bombyliidae | Bombilide11 | B11 | 13 | 1 |
| Lepidoptera | Pieridae | *Ascia josephina* | Aj | 9 | 0.7 |
| Diptera | Bombyliidae | Bombilide10 | B10 | 9 | 0.7 |
| Diptera | Muscidae | Muscidae | M | 8 | 0.6 |
| Lepidoptera | Lycaenidae | Lycaenidae4 | L10 | 7 | 0.5 |
| Lepidoptera | Lycaenidae | Lycaenida3 | L9 | 7 | 0.5 |
| Diptera | Bombyliidae | *Crysntrax cypris* | B5 | 6 | 0.4 |
| Hymenoptera | Megachilidae | *Megachile zaptlana* | Mh | 5 | 0.4 |
| Lepidoptera | Pieridae | *Prysitas spp* | L1 | 3 | 0.2 |
| Lepidoptera | Riodinidae | Rionidae3 | L12 | 3 | 0.2 |
| Lepidoptera | Nymphalidae | Nymphalide1 | L2 | 3 | 0.2 |
| Hymenoptera | Apidae | *Xylocopa mexicanorum* | Xm | 2 | 0.1 |
| Hymenoptera | Vespidae | *Zethus spp* | Av9 | 1 | 0.1 |
| Lepidoptera | Nymphalidae | *Agraullis vanillae* | Ava | 1 | 0.1 |
| Hymenoptera | Halictidae | *Halictide spp* | Ha | 1 | 0.1 |
| Lepidoptera | Lycaenidae | Lycaenidae2 | L7 | 1 | 0.1 |
| Diptera | Syrphidae | Syrphidae1 | S1 | 1 | 0.1 |
| **Cancunito** |  |  |  |  |  |
| Hymenoptera | Apidae | *Apis mellifera* | Am | 862 | 61.9 |
| Diptera | Bombyliidae | *Exoprosa spp* | B1 | 220 | 15.8 |
| Lepidoptera | Pieridae | *Ascia josephina* | Aj | 98 | 7 |
| Lepidoptera | Pieridae | *Ascia monuste* | Amo | 61 | 4.4 |
| Diptera | Bombyliidae | *Crysntrax cypris* | B5 | 43 | 3.1 |
| Diptera | Bombyliidae | *Crysntrax disppar* | B7 | 15 | 1.1 |
| Diptera | Bombyliidae | Bombilide13 | D2 | 15 | 1.1 |
| Lepidoptera | Hespperiidae | *Cymaenes trebius* | Lp | 14 | 1 |
| Hymenoptera | Vespidae | *Zethus spp* | Av9 | 12 | 0.9 |
| Lepidoptera | Nymphalidae | *Agraullis vanillae* | Ava | 10 | 0.7 |
| Hymenoptera | Halictidae | *Lassoiglosum spp* | La | 8 | 0.6 |
| Hymenoptera | Vespidae | *Micodynerus spp* | Av16 | 6 | 0.4 |
| Hymenoptera | Megachilidae | *Megachile zaptlana* | Mh | 6 | 0.4 |
| Hymenoptera | Vespidae | Vesppide2 | Av1 | 5 | 0.4 |
| Diptera | Bombyliidae | Bombilide6 | B6 | 5 | 0.4 |
| Diptera | Bombyliidae | Bombilide15 | D4 | 3 | 0.2 |
| Lepidoptera | Lycaenidae | Lycaenidae4 | L10 | 3 | 0.2 |
| Lepidoptera | Nymphalidae | *Junonia scenia* | Js | 2 | 0.1 |
| Lepidoptera | Hespperiidae | *Urbanus doryssus* | L6 | 2 | 0.1 |
| Hymenoptera | Vespidae | Vesppide11 | Av10 | 1 | 0.1 |
| Diptera | Bombyliidae | *Geron spp* | D3 | 1 | 0.1 |
| Lepidoptera | Pieridae | *Phoebis spp* | L11 | 1 | 0.1 |
| **Charcas** |  |  |  |  |  |
| Hymenoptera | Vespidae | *Micodynerus spp* | Av16 | 110 | 21.8 |
| Hymenoptera | Halictidae | *Lassoiglosum spp* | La | 51 | 10.1 |
| Diptera | Muscidae | Muscidae | M | 39 | 7.7 |
| Diptera | Bombyliidae | Bombilide11 | B11 | 31 | 6.2 |
| Hymenoptera | Megachilidae | *Megachile zaptlana* | Mh | 31 | 6.2 |
| Hymenoptera | Apidae | *Apis mellifera* | Am | 29 | 5.8 |
| Lepidoptera | Nymphalidae | *Junonia scenia* | Js | 27 | 5.4 |
| Diptera | Bombyliidae | *Crysntrax cypris* | B5 | 25 | 5 |
| Hymenoptera | Vespidae | Vesppide5 | Av4 | 18 | 3.6 |
| Hymenoptera | Vespidae | *Polistes* spp1 | C1 | 16 | 3.2 |
| Diptera | Bombyliidae | *Crysntrax disppar* | B7 | 15 | 3 |
| Diptera | Bombyliidae | *Geron spp* | D3 | 15 | 3 |
| Lepidoptera | Lycaenidae | Lycaenidae4 | L10 | 15 | 3 |
| Hymenoptera | Vespidae | *Polistes spp* 4 | C3 | 9 | 1.8 |
| Diptera | Bombyliidae | Bombilide15 | D4 | 9 | 1.8 |
| Hymenoptera | Vespidae | *Zethus spp* | Av9 | 8 | 1.6 |
| Hymenoptera | Vespidae | Vesppide7 | Av6 | 7 | 1.4 |
| Diptera | Bombyliidae | Bombilide16 | D5 | 6 | 1.2 |
| Diptera | Bombyliidae | Bombilide19 | D8 | 6 | 1.2 |
| Lepidoptera | Lycaenidae | Lycaenidae1 | L3 | 6 | 1.2 |
| Lepidoptera | Hespperiidae | Hespperidae | L8 | 6 | 1.2 |
| Lepidoptera | Hespperiidae | *Cymaenes trebius* | Lp | 6 | 1.2 |
| Hymenoptera | Vespidae | *Polistes spp* 3 | C2 | 4 | 0.8 |
| Diptera | Bombyliidae | Bombilide12 | D1 | 4 | 0.8 |
| Lepidoptera | Pieridae | *Ascia josephina* | Aj | 3 | 0.6 |
| Lepidoptera | Pieridae | *Ascia spp* | Aspp | 3 | 0.6 |
| Hymenoptera | Vespidae | Vesppide13 | Av12 | 2 | 0.4 |
| Lepidoptera | Pieridae | *Ascia monuste* | Amo | 1 | 0.2 |
| Diptera | Bombyliidae | Bombilide9 | B9 | 1 | 0.2 |
| Diptera | Syrphidae | Syrphidae1 | S1 | 1 | 0.2 |
| **Punta Meco** |  |  |  |  |  |
| Hymenoptera | Apidae | *Apis mellifera* | Am | 1443 | 78.1 |
| Lepidoptera | Hespperiidae | *Cymaenes trebius* | Lp | 82 | 4.4 |
| Hymenoptera | Vespidae | Vesppide14 | Av13 | 73 | 4 |
| Hymenoptera | Vespidae | *Polistes spp*1 | C1 | 32 | 1.7 |
| Hymenoptera | Vespidae | *Micodynerus spp* | Av16 | 28 | 1.5 |
| Hymenoptera | Vespidae | *Polistes spp* 2 | Pol | 27 | 1.5 |
| Hymenoptera | Halictidae | *Lassoiglosum spp* | La | 22 | 1.2 |
| Hymenoptera | Vespidae | *Zethus spp* | Av9 | 15 | 0.8 |
| Diptera | Muscidae | *Muscidae* | M | 15 | 0.8 |
| Diptera | Bombyliidae | *Exoprosa spp* | B1 | 14 | 0.8 |
| Hymenoptera | Vespidae | Vesppide7 | Av6 | 12 | 0.6 |
| Diptera | Bombyliidae | *Crysntrax cypris* | B5 | 12 | 0.6 |
| Hymenoptera | Vespidae | Vesppide13 | Av12 | 9 | 0.5 |
| Lepidoptera | Pieridae | *Ascia monuste* | Amo | 8 | 0.4 |
| Diptera | Bombyliidae | *Crysanthrax editilius* | B3 | 8 | 0.4 |
| Diptera | Bombyliidae | *Geron spp* | D3 | 7 | 0.4 |
| Hymenoptera | Vespidae | Vesppide6 | Av5 | 5 | 0.3 |
| Diptera | Bombyliidae | *Crysntrax disppar* | B7 | 5 | 0.3 |
| Lepidoptera | Nymphalidae | *Junonia scenia* | Js | 4 | 0.2 |
| Lepidoptera | Pieridae | *Ascia josephina* | Aj | 3 | 0.2 |
| Diptera | Bombyliidae | Bombilide6 | B6 | 3 | 0.2 |
| Diptera | Bombyliidae | Bombilide15 | D4 | 3 | 0.2 |
| Lepidoptera | Nymphalidae | Nymphalide1 | L2 | 3 | 0.2 |
| Hymenoptera | Megachilidae | *Megachile zaptlana* | Mh | 3 | 0.2 |
| Lepidoptera | Pieridae | *Ascia spp* | Aspp | 2 | 0.1 |
| Diptera | Bombyliidae | Bombilide12 | D1 | 2 | 0.1 |
| Diptera | Bombyliidae | Bombilide19 | D8 | 2 | 0.1 |
| Lepidoptera | Hespperiidae | Hespperidae | L5 | 2 | 0.1 |
| Hymenoptera | Vespidae | Vesppide11 | Av10 | 1 | 0.1 |
| Diptera | Bombyliidae | Bombilide11 | B11 | 1 | 0.1 |
| Lepidoptera | Hespperiidae | Hespperidae | L8 | 1 | 0.1 |
| Diptera | Syrphidae | Syrphidae1 | S1 | 1 | 0.1 |
