# Supplementary material for: The role of alien species on plant-floral visitor network structure in invaded communities
Source: PLoS One. 2019 Nov 8;14(11):e0218227. doi: 10.1371/journal.pone.0218227 (PMC6839871; doi:10.1371/journal.pone.0218227)
Supplement: S3 Table — Complementary plant-pollinator interaction network metrics in nine sites across three geographic regions in the north coast of Yucatan. Significant values of connectance are shown in bold (P<0.05). Sites are ordered according to proportion of alien flower abundance (see Table 1). (DOCX) [file pone.0218227.s003.docx]

Table S3. Complementary plant-pollinator interaction network metrics in nine sites across three geographic regions in the north coast of Yucatan. Significant values ​​of connectance are shown in bold (P<0.05). Sites are ordered according to proportion of alien flower abundance (see Table 1).

| Sites | Links per species | Pairwise interactions | Connectance |
| --- | --- | --- | --- |
| Chapo 1 | 1.61 | 69 | **0.19** |
| Playa Maya | 2.34 | 71 | **0.25** |
| Chapo 2 | 1.71 | 85 | **0.17** |
| Telchac | 2.06 | 103 | **0.20** |
| Cancunito | 1.70 | 51 | **0.29** |
| Punta Meco | 1.84 | 85 | **0.19** |
| Sisal | 1.47 | 56 | **0.19** |
| Charcas | 1.73 | 71 | **0.22** |
| Chabiahu | 2.02 | 91 | **0.19** |
