# Supplementary figures and images for: The role of alien species on plant-floral visitor network structure in invaded communities
Source: PLoS One. 2019 Nov 8;14(11):e0218227. doi: 10.1371/journal.pone.0218227 (PMC6839871; doi:10.1371/journal.pone.0218227)

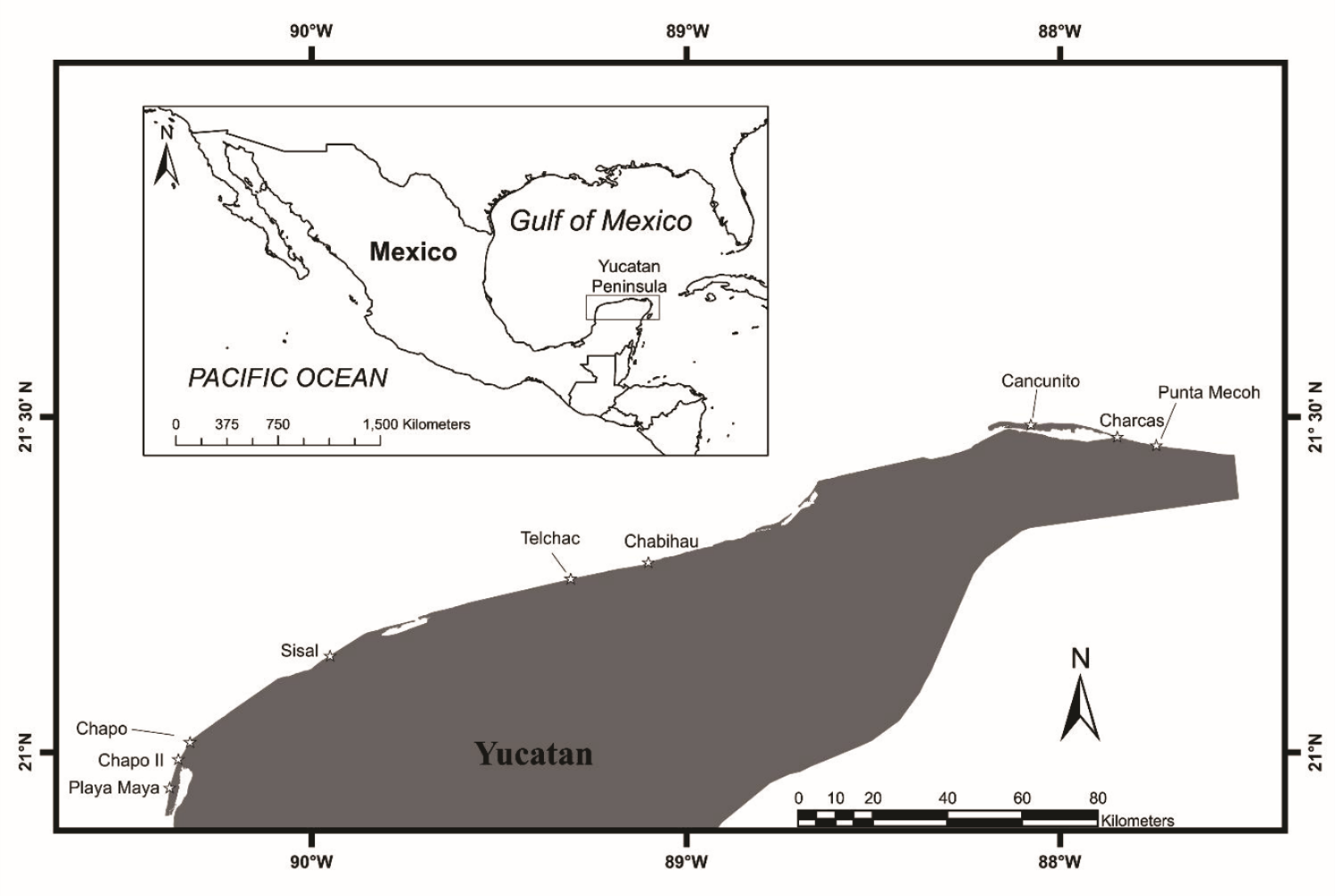


S1 Figure. Location of the nine study sites along the north of the Yucatan Peninsula, Mexico.

Supplement: S1 Fig — (DOCX) [file pone.0218227.s004.docx]
