# Supplementary material for: The role of alien species on plant-floral visitor network structure in invaded communities
Source: PLoS One. 2019 Nov 8;14(11):e0218227. doi: 10.1371/journal.pone.0218227 (PMC6839871; doi:10.1371/journal.pone.0218227)
Supplement: S2 Fig — Size effects (CI 95%) for overall specialization, nestedness, modularity and robustness contrasting different scenarios with intact networks, (a) “Aliens removed”, (b) “natives removed”, and (c) “mixed removal”. (DOCX) [file pone.0218227.s005.docx]

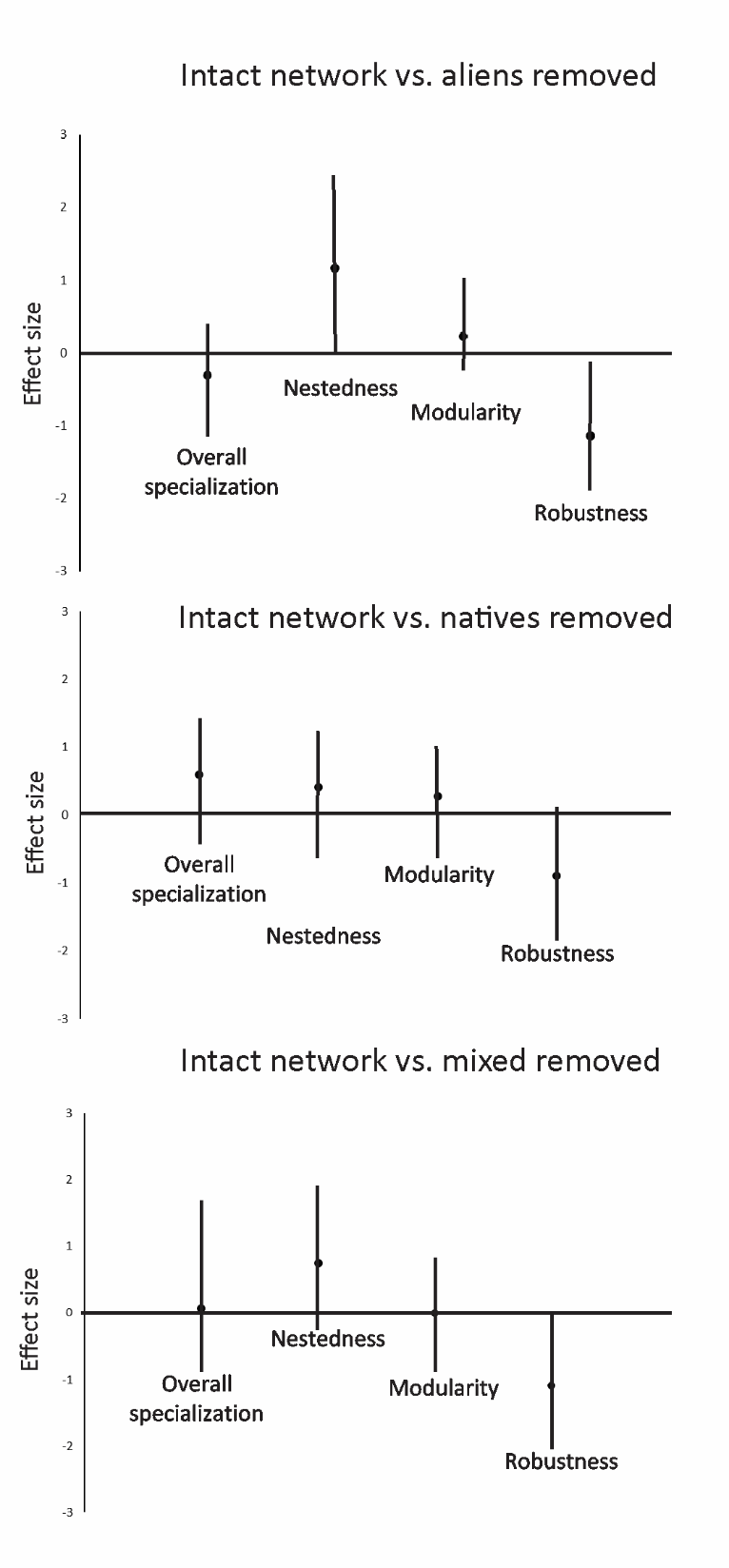


S2 Figure. Size effects (CI 95%) for overall specialization, nestedness, modularity and robustness contrasting different scenarios with intact networks, (a) “Aliens removed”, (b) “natives removed”, and (c) “mixed removal”.
